# Synthesis and electrochemical performance of α-Al_2_O_3_ and M-Al_2_O_4_ spinel nanocomposites in hybrid quantum dot-sensitized solar cells

**DOI:** 10.1038/s41598-022-21186-4

**Published:** 2022-10-11

**Authors:** Sawsan A. Mahmoud, Moustafa E. Elsisi, Asmaa F. Mansour

**Affiliations:** 1 Egyptian Petroleum Research Institute, Nasr City, Cairo 11727 Egypt; 2 Physics Department, Faculty of Science, Zagazig University, Zagazig, Egypt

**Keywords:** Renewable energy, Electrical and electronic engineering

## Abstract

The aim of this study is to describe the performance of the aluminum oxide nanoparticle and metal aluminate spinel nanoparticle as photo-anodes in quantum dot photovoltaic. By using a sol–gel auto combustion method, Al_2_O_3_ NPs, CoAl_2_O_4_, CuAl_2_O_4_, NiAl_2_O_4_, and ZnAl_2_O_4_ were successfully synthesized. The formation of Al_2_O_3_ NPs and MAl_2_O_4_ (M=Co, Cu, Ni, Zn) nanocomposite was confirmed by using several characteristics such as XRD, UV–Vis, FTIR, FE-SEM, and EDX spectra. The XRD shows that the CoAl_2_O_4_ has a smaller crystallite size (12.37 nm) than CuAl_2_O_4,_ NiAl_2_O_4_, and ZnAl_2_O_4_. The formation of a single-phase spinel structure of the calcined samples at 1100 °C was confirmed by FTIR. Our studies showed that the pure Al_2_O_3_ NP_s_ have a lower energy gap (1.37 eV) than synthesized MAl_2_O_4_ under UV–Vis irradiation. Due to the well separation between the light-generated electrons and the formed holes, the cell containing ZnAl_2_O_4_ nanocomposite with CdS QDs has the highest efficiency of 8.22% and the current density of 22.86 mA cm^−2^, while the cell based on NiAl_2_O_4_ as a photoelectrode, six cycles of CdS/ZnS QDs, and P-rGO as a counter electrode achieved the best (PCE) power conversion efficiency of 15.14% and the current density of 28.22 mA cm^−2^. Electrochemical impedance spectroscopy shows that ZnAl_2_O_4_ and NiAl_2_O_4_ nanocomposites have the highest life times of the photogenerated electrons (*τ*_*n*_) of 11*10^−2^ and 96*10^−3^ ms*,* respectively, and the lowest diffusion rates (K_eff_) of 9.09 and 10.42 ms^−1^, respectively.

## Introduction

Alpha-alumina and metal aluminate spinel with the formula M-Al_2_O_4_, where M introduces a divalent metal ion, have attracted great attention for several applications due to their thermal stabilities, high chemical resistance and mechanical resistance, high quantum yields with hydrophobic qualities, high surface area, and low acidic surface^1–10^. They have been widely used as pigments, sensors, photocatalysts, magnetic, refractory, optical, and materials electrodes, involving lubricant additives^11–15^. It has been known that the synthesis method can influence the crystallinity, purity, surface area, morphology, and particle size of nanosynthesized materials MAl_2_O_4_, which has a respectable impact on their catalytic and optical properties^16–18^. MAl_2_O_4_ can be used with a variety of techniques including solvothermal method, co-precipitation synthesis, sol–gel method, solid-state reactions, hydrothermal method, microwave-assisted, polymeric precursor synthesis, and hydrothermal method^16–30^. Either method requires specialized accommodation and has a respectable cost, while further disadvantages include the shortage of homogeneity and low surface area of product^5–10,16–18,31–33^. The generation of the homogeneous high-purity nanoparticles has been demonstrated by the sol–gel auto combustion technique with swift heating and short reaction time^11,12,34^. The sol–gel auto combustion synthesis method combines chemical sol–gel and combustion processes, representing a swift, attainable technique with low energy costs, and is perfect for the synthesis of materials based on metal oxides. Several organic compounds can be utilized as the fuel, but these have been imitatively fixed to citric acid, urea, glycine, and tartaric acid^7,12^. Graphene is an intriguing material with a novel two-dimensional skeleton composed of a single monomolecular layer of sp^2^-hybridized carbon atoms^1,4^. Graphene has excellent properties in many areas of technology and science due to its unique properties^5,6^, including superior electronic^6–8^, mechanical, and thermodynamic properties^9,10^. Graphene has a broad domain of applications such as field-effect transistors (FET), transparent conductive films, energy storage devices, water purification, and sensors due to its elegant physical and chemical properties^16–18,31,32^. P-rGO is a carbon material that shows optical, chemical, and electrical characteristics similar to those of graphene because it is based on its framework^35^. In 1958, Hummers and Offman have well-educated a method for the synthesis of P-rGO^36–41^. This method uses H_2_SO_4_ to peel graphite with NaNO_3_ and KMnO_4_ as the oxidizing agents for graphite. The method of Hummer has some features compared to that of Brodie and Staudenmaier. Firstly, KMnO_4_ is a strong oxidant that aids in hastening the reaction so that the synthesis can be finished in a few hours. Secondly, the chlorate is not available, removing the probability of a ClO_2_ eruption. Thirdly, the exchange of fumigation with NaNO_3_ eliminates the acid haze generated by HNO_3_^15^. To the best of our knowledge, there have been no studies comparing the effects of NiAl_2_O_4_, CuAl_2_O_4_, and ZnAl_2_O_4_ spinel aluminates, which were produced by the same synthetic method, on the performance of QDSSCs. Solar cells sensitized by quantum dots (QDSSCs) have attracted massive awareness in recent years, owing to their easy-fabrication procedure, low-cost, adjustable bandgap, and high theoretically mentioned power conversion efficiency (PCE) of up to 44%^42^. The photoelectrochemical technicality of QDSSCs has the same behavior as dye-sensitized solar cells (DSSCs), in which the solar cell is sensitized by the QDs instead of the dye molecules as a light absorber layer in QDSSCs^43^. A QDSSC is typically composed of a quantum dot sensitised photoanode film, an electrolyte containing a redox couple (i.e., S_2_/Sx_2_), and a counter electrode (CE) (i.e., Pt, and Cu_2_S)^42–44^. In spite of these qualities, however, the performance of the photovoltaic cells of most QDSSCs is much lower than that acquired from DSSCs. To date, much of the research work has concentrated on enhancing all the elements in QDSSCs. Since one of the essential reasons for low efficiency is the counter electrode interfaces, particular research efforts have settled on the expansion of congruous CEs for achieving simultaneous high efficiency and stable cells.

In the published literature, graphene-TiO_2_ nanocomposite has a large absorption band of visible light. The graphene-TiO_2_ nanocomposite photoanode has been deposited onto FTO using the doctor blade method as a photoanode to obtain a negative capacitance. Photovoltaic cells (PV) currently have a low efficiency in converting light into electrical power. Despite the superposition of several PV cells that convert more than 41% of incident light power to electricity and several semiconductor materials having an efficiency lower than 21% as a result of the great use of energy in the industry, these PVs can only convert one third of the light power received. This efficiency is limited by some physical properties of the materials, such as the Joule losses. The challenge of the scientific community is to prevent the leakage of electrons to energy. Most of the PV cells are prepared from semiconductor materials because of their very special properties. It can absorb photons of a well-defined wavelength to generate free electrons, which then gives an electric current. Several studies have shown that the GO can generate many pairs of electron-holes by absorbing one photon and relaxing the primary pair of electron-holes excited by illumination. Instead of losing excess excitation energy by the joule effect or phonon form, graphene is a suitable material to create hot charge carriers by transferring the excess energy to other carriers. The power conversion efficiency (η) and the short current Isc increased with the graphene amount due to the intrinsic properties of this material. The efficiency of the process is correlated to the energy lost by the joule effect or phonon form. The efficiency can be increased if the excited charge carriers use their excess energy to produce electron–hole pairs through an interaction carrier-carrier or a diffusion process, instead of losing energy in the form of heat and phonons. In particular, the best performing solar cells employing rGO exhibited power conversion efficiency (PCE) of up to 18.13%, while the control device without rGO delivered a maximum efficiency of 17.26%. An effective and low-cost approach has been proposed for the fabrication of reduced graphene oxide (rGO) solar cells. The hybrid composite of rGO has been synthesized by using a low-cost chemical method and deposited on a glass substrate by a doctor-blading process for application in heterojunction solar cells. The morphology, crystal phase, surface functional groups, and optoelectronic properties of the heterostructure have been explored. The J-V characteristics of the fabricated heterojunction cell show a power conversion efficiency of 4.35% (V_oc_ = 0.51 V, J_sc_ = 14.47 mA cm^−2^, and fill factor (FF) = 51.60) due to the enhanced conductivity and charge transfer efficiency of the hybrid semiconductor. This simple approach assures the low-cost mass production of heterojunction solar cells^45–49^. Kafle et al. reported that the best performing solar cells employing rGO exhibited power conversion efficiency (PCE) of up to 18.13% in a perovskite cell composed of rGO as a counter electrode, while the control device without rGO delivered a maximum efficiency of 17.26%^46^. Kadhim et al., used the cell composed of TiO_2_ as a photoanode, CdS QD_s_, polysulphide electrolyte, and rGO as a counter electrode to give a PCE of 4.35%^47^. Mnasri et al., used the quantum dot sensitized solar cell composed of GO-TiO_2_ nanocomposites as a photoanode, five cycles of CdS QD_s_, polysulphide electrolyte and Pt as a counter electrode to give a PCE of 0.3%^48^.

In this work, for the first time, we have grown first-step, electrocatalytically active, highly stable CoAl_2_O_4_, NiAl_2_O_4_, CuAl_2_O_4_, and ZnAl_2_O_4_ spinel nanostructures by a sol–gel auto combustion technique as the working electrode in hybrid structures of ZnS/CdS quantum dots-based solar cells. The morphology of the surface and optical behavior of the aluminate spinels (MAl_2_O_4_ (M=Ni, Cu, Zn)) have been inspected by XRD, FT-IR, SEM, and UV–visible spectroscopy (UV–Vis). The effect of hybrid CdS QDSSC/ZnS QDs was also studied. The QDSSCs, which contain ZnAl_2_O_4_ as a photoanode, six cycles of CdS QDs as a photosensitized material, polysulphide electrolyte, and rGO as a counter electrode, gave a PCE of 8.22%. The cell contains a NiAl2O4 as a photoanode, twelve cycles of ZnS and CdS QDs, polysulphide electrolyte, and rGO as a counter electrode, given a PCE of 15.14%.

## Experimental methods

*Materials* Aluminum Nitrate (Al_2_(NO_3_)_3_.9H_2_O (97%, Aldrich), Zinc Acetate Zn(CH_3_COO)_2_ (97%, Bio Chem), Sodium Sulfide Na_2_S **(**98%, Alpha chemical), Ethanol absolute (99%, Bio Chem), Nitric acid HNO_3_ (Adwic), sulfuric acid H_2_SO_4_, (Dongwoo fin chem., 95–97%), Triton-X100 (97%, Aldrich), Extra pure graphite powder (12.0 g/ mole, 99.5%, Aldrich), Sulfur Powder (Adwic), Potasium Chloride (Advent), FTO (Fluorine-dopped SnO_2_) conductive glass (Aldrich), Nickle Chloride (NiCl_2_.6H_2_O), Cobalt Nitrate (Co(NO_3_)_2_) and Cupper Chloride (CuCl_2_.2H_2_O) from Adwic, Potassium Permanganate (KMnO_4_, Sigma Aldrich 97%), Hydrogen Peroxide (H_2_O_2_, Dongwoo fin chem., 30%).

### Synthesis of aluminum oxide (Al_2_O_3_) NP_s_

The auto-combustion sol–gel method used for the synthesis of Al_2_O_3_ nanostructured, Al_2_ (NO_3_)_3_.9H_2_O was used as the precursor. Solution A contains a desired amount of aluminum nitrate nonahydrate and was dissolved in ethanol to prepare a 0.1 M of Al_2_ (NO_3_)_2_ ethanoic solution by stirring for about 2 h using a magnetic stirrer. Solution B contains a 30% ammonia solution. The gel of aluminum oxide was formed by adding the 30% ammonia solution dropwise to the aqueous aluminum nitrate solution under vigorous stirring for one h. This gel was left for 30 h at room temperature for maturation time and dried at 200 °C for 24 h. The resulting gel was annealed in a furnace for 4 h at 1100ºC^11^.

### Synthesis of metal doped aluminum oxide (M-Al_2_O_4_)

The M-Al_2_O_4_ spinel nanostructures were prepared by the sol–gel auto combustion method. Stoichiometric amounts of Al_2_ (NO_3_)_3_.9H_2_O and metal nitrate were dissolved in absolute ethanol to obtain a 0.2 M solution. Then, a convenient amount of DEA was added as fuel to the solution. The mixture was heated to 200 °C under continuous stirring until all of the ethanol had evaporated and the process took place. The mixed solution mutated into a condensed gel, which self-ignited to produce a pearly yellow powder for ZnAl_2_O_4_^16,26,50–52^, a dark powder for CoAl_2_O_4_, a brown powder for CuAl_2_O_4_^21,24,25,30^ and a grey powder for NiAl_2_O_4_^19,20,22,23^. Finally, the as-synthesized powders were annealed in the air at 1100 °C for 4 h^16^.

### Synthesis of partially reduced-graphene oxide (P-rGO)

Partially-reduced graphene oxide (P-rGO) was synthesized from the graphite powder according to the modified Hummers and Offman method^37–39^. In a solution containing 50 ml of concentrated H_2_SO_4_ and 50 ml of concentrated HNO_3_, the extra pure graphite powder (2.0 g) was pre-oxidized by slowly adding it to the mixture and stirring at 80 °C for 4 h. The mixture was cooled down to room temperature and then washed by de-ionized water until the pH value was neutral (equal to 7.0), followed by drying at 40 °C overnight. The resultant pre-oxidized graphite was dispersed into concentrated H_2_SO_4_ in a cold reaction vessel, which was kept in an ice bath and stirred. 10 g of KMnO_4_ was slowly added to it. During the addition, the temperature was kept below 10 °C. The mixture was stirred at 35 °C for 2 h until the solution became gelled and turned a brownish gray in color. Then 250 ml of de-ionized water was added and the temperature was put forward to 100 °C for 15 min, followed by adding 700 ml of de-ionized water and 30 ml of H_2_O_2_ to the mixture, stirred for 1 h. The solid products were composed from the solution after 12 h and washed with 5% HCl until sulphate ions were no longer detectable with BaCl_2_. Then the solid products were re-dispersed in de-ionized water five times to eliminate any impurities. Finally, the resultant sediment was dried at 60 °C for 4 h in an oven to yield the partially reduced graphene oxide P-r (GO)^15,35–39^. Fig. S1 shows the steps of rGO preparation step by step.

### Fabrication of Al_2_O_3_NP_s_ nanoparticle and M-Al_2_O_4_ nanocomposites films

Both Al_2_O_3_ nanoparticle and M-Al_2_O_4_ nanocomposite films with a thickness of 0.052 mm were prepared and used for solar cell testing. The samples of Al_2_O_3_NP and M-Al_2_O_4_ nanocomposites were deposited onto conductive Fluorine-tin-oxide (FTO) glass substrates by the doctor-blading method using a dispersion of 0.2 g of Al_2_O_3_ powder in ethanol solution and the same of 0.2 g of CoAl_2_O_4_, CuAl_2_O_4_, NiAl_2_O_4_ and ZnAl_2_O_4_. Before spreading the film, the surfaces of the FTO substrates were cleaned for 30–60 min using an ultrasonicator. The substrates were then dried in air. The FTO boundaries of each substrate were covered with scotch tape to control the area of the film. The powder was first strewn in Triton-X100 and ethanol, and then the pending powder was added as drops in the midst of the substrate and prevalence to form a thick film to produce the film. The film was dried in the air for 30 min and then calcined at 300 °C for 5 min^46–53^.

### Preparation of photoelectrode with different quantum dots to form hybrid structures (CdS/ZnS)

The photo electrode was sensitized with the CdS QDs, which act as light absorbers by the Successive Ionic Layer Adsorption and Reaction (SILAR) method. To grow CdS QDs, the semiconductor film was immersed in a 0.2 M Cadmium nitrate (CdNO_3_)_2_ aqueous solution for 2 min as a Cd^+2^ source, then rinsed in ethanol to remove surplus ions and dried on a hot plate at 60 °C for 1 min. Then, the film was dipped into a 0.2 M Na_2_S aqueous solution for another 2 min to allow S^2^ to react with the pre-adsorbed Cd^2+^ leading to the formation of CdS QDs. Swiping the film in methanol and drying at 60 °C for 1 min removed the loosely bound S^2−^ions. On the other hand, the hybrid structures of ZnS/CdS QDs are prepared by the same method (SILAR) by the growth of ZnS QDs. The semiconductor film was immersed in a 0.2 M Zn (CH_3_COO)_2_ aqueous solution for 2 min before being rinsed with ethanol to remove the excess ions and dried for 1 min on a hot plate at 60 °C. Then, the film was dipped into a 0.2 M Na_2_S aqueous solution for another 2 min to allow S^2^ to react with the pre-adsorbed Zn^2+^, leading to the formation of ZnS QDs. The loosely bound S^2−^ions were removed by swiping the film in methanol and drying it at 60 °C for 1 min. Second, a semiconductor and ZnS QDs film were dipped into a 2 M Cd (NO_3_)_2_ aqueous solution as a Cd^+2^ source. Then, the film was dipped into a 0.2 M Na_2_S aqueous solution for another 2 min to allow S^2−^to react with the pre-adsorbed Cd^2+^, leading to the formation of hybrid structures of CdS QDs and ZnS QDs. The loosely bound S^2−^ions were removed by rinsing the film in methanol and then the film was dried at 60 °C for 1 min. To increase the crystallinity of QDs, the films with different quantum dot depositions were calcined at 300 °C for 5 min^45^.

### Preparation of electrolyte

A polysulfide electrolyte was prepared and used. The electrolyte is composed of 2 M Na_2_S, 2 M sulphur powder, and 0.2 M KCl mixed solution of methanol and deionized water with a volume ratio of 7:3 respectively. Electrolytes were deposited between the top of the QDs-coated anode and the counter electrode^46^.

### Assembling the CdS QDs sensitized solar cell

The two electrodes, photo and counter electrodes, were clipped together facing each other using two clips, and the drops of electrolyte solution could then be put at the edges of the plates. The two warp clips are off and on, opening and closing while in place. An electrolyte is attracted to the space between the electrodes by capillary action. The light source was directed to each solar cell device, allowing light to penetrate the solar cell to the CdS QDs and CdS/ZnS adsorbed onto the Al_2_O_3_ NP and M-Al_2_O_4_ spinel film electrodes shown in Fig. S2.

### Photoelectrochemical efficiency

The performance of the solar cell was measured using strained solar illumination with a 10.4 mW/cm^2^ light output. The short-circuit current and open-circuit voltage are measured by using photocell software, as well as calculating the cell efficiency. The light intensity source was measured by a radiometer (international light 1700). All the J-V curves of the solar cells were obtained in the dark and under illumination. The J-V characteristics as a function of incident light intensity were used to obtain the open-circuit voltage (Voc), short-circuit current density (Jsc), the maximum voltage point (Vmax), and the maximum current density point (Jmax).

### Methods of analysis

Different techniques were used to examine the surface of prepared materials.

The X-ray diffraction patterns were reported using a Pan Analytical Model X 'Pert Pro, which was fitted with CuKα radiation (α = 0.1542 nm), a Ni-filter, and a general area detector. A 40 kV accelerating voltage and a 40 mA emission current were utilized. The diffractograms were measured in the 2θ range from 0.5 to 70 °.

The Fourier transform infrared spectroscopy (FT-IR) of the prepared samples was measured using the KBr technique adopted by the Nicolet Is-10 FT-IR spectrophotometer (Thermo Fisher Scientific). The KBr technique was conducted roughly in a quantitative manner for all samples, since the sample weight and that of KBr were both held constant.

A Field Emission Scanning Electron Microscope (FE-SEM) is used to examine the material's composition, particle size, and shape. For the samples prepared, a 30 kV acceleration voltage operating on a JSM-7500F electron microscope was reported.

Optical absorption spectra of the sample were analyzed using Ultraviolet–Visible absorption spectroscopy (Spectro UV–Vis 2800, United States).

Dielectric studies of Al_2_O_3_ NP_s_ and M-Al_2_O_4_ NC_s_ were executed as thin films on conducting glass (FTO) of dimension (6.25 cm^2^ surface area and a 0.052 mm thickness) for every sample to serve as electrodes during the measurements by a standard two-probe technique using an impedance analyzer (IM3570, Japan).

## Results and discussion

### XRD analysis

**Figure 1:**
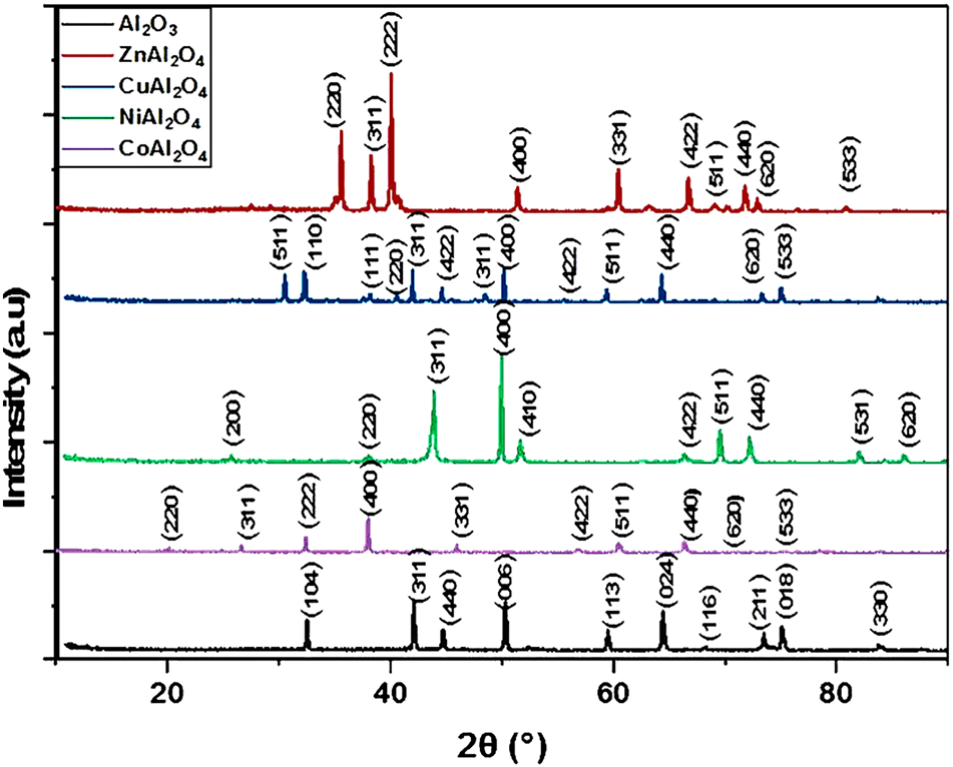
XRD spectra of Al2O3 NPs, ZnAl2O4, CoAl2O4, NiAl2O4, and CuAl2O4 nanocomposites at 1100 °C.

The X-ray diffraction pattern (XRD) for Al_2_O_3_ NPs, ZnAl_2_O_4_, NiAl_2_O_4_, CoAl_2_O_4_, and CuAl_2_O_4_ spinel is shown in Fig. 1. The XRD of highly crystalline α-Al2O3 shows the characteristic peaks at 35.17°, 43.379°, 45.47°, 52.55°, 57.516°, 66.513°, 68.218°, 73.42°, 75.16° and 83.778° corresponding to the crystal planes, (104), (311), (400), (006), (113), (024), (116), (211), (018) and (214), which confirmed the formation of Al_2_O_3_ NPs in a pure single hexagonal phase according to JCPDS Card No. 42-1468. Figure 3 shows the cubic spinel structure of NiAl_2_O_4_, which exhibited diffraction peaks at 25.73°, 38.006°, 43.87°, 49.593°, 51.626°, 66.306°, 67.59°, 72.146°, 81.966° and 86.046° corresponding to the crystal planes, (200), (220), (311), (400), (410), (422), (511), (440), (531) and (620), which confirmed the formation of a pure single phase of NiAl_2_O_4_, in agreement with JCPDS No. 10-0339^40,41,54,55^. The strong and sharp diffraction peaks indicate that NiAl_2_O_4_ has a long-range ordered structure. Figure 1 shows a single phase of CuAl_2_O_4_ cubic spinel (JCPDS No. 33-0448) calcined at 1100 °C^21,24,25,35,40^. The Figure also depicts the characteristic CuAl_2_O_4_ peaks at 23.66°, 25.406°, 31.305°, 35.103°, 37.73°, 43.43°, 48.65°, 52.50°, 57.62°, 66.46°, 68.138°, 73.29 and 76.83° corresponding to the crystal planes, (511), (110), (111), (220), (311), (422), (311), (400), (422), (511), (440), (620) and (533). The formed peaks are congruent to the formation of ZnAl_2_O_4_ (JCPDS No. 05-0669)^41,54,55^ in a single phase structure with characteristic peaks at 35.599°, 38.28°, 40.079°, 51.37°, 60.36°, 66.76°, 69.12°, 71.78°, 72.86° and 80.89° corresponding to the crystal planes, (220), (311), (222), (400), (331), (422), (511), (440), (620) and (533). CoAl_2_O_4_, the characteristic peaks appear at 19.034°, 25.46°, 31.28°, 36.85°, 44.84°, 55.67°, 59.31°, 65.71°, 74.15°and 77.33° corresponding to the crystal planes, (220), (311), (222), (400), (331), (422), (511), (440), (620) and (533) (JCPDS No. 44-0160). In addition, the average crystallite size (D) of spinel aluminate nanostructures can be predestined from the full width at half maximum of the strongest diffraction peak by applying the Debye–Scherrer equation, Eq. (1). Table S1 shows the crystallite size, thickness of samples, and band gap energy.

1 $$ {\text{D}} = \frac{{0.9{\uplambda }}}{{{\upbeta }\cos {\uptheta }}} $$

### FTIR analysis

**Figure 2:**
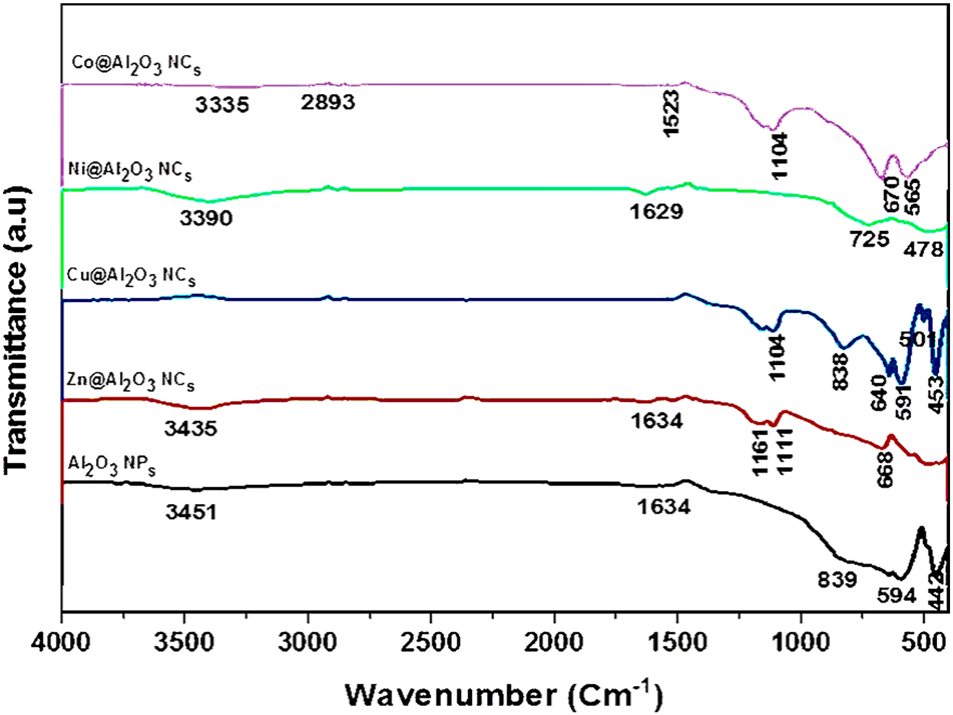
FTIR spectra of Al2O3 NPs, ZnAl2O4, CoAl2O4, NiAl2O4, and CuAl2O4 nanocomposites at 1100 °C.

Figure 2 shows the typical transmittance FT-IR spectra of Al_2_O_3_ NP_s,_ ZnAl_2_O_4_, NiAl_2_O_4_, CoAl_2_O_4_, and CuAl_2_O_4_ spinel oxides calcined at 1100 ℃ as a set of transmission peaks in the range of 400–4000 cm^−1^. The functional groups of aluminum oxide NPs and metal aluminate spinel structures were inspected by FT-IR analysis. The stretching mode of Al–O and the Al–O–Al bending mode in the spectrum of Al_2_O_3_ NPs appear at the peaks of 594 and 1598 cm^−1^, respectively. A band at 3451 cm^−1^ representing the –OH species was detected for the Al–OH bond formation. A small peak at 1634 cm^−1^ was attributed to the H–O–H bending vibration characteristic peak. This confirms the existence of water molecules absorbed on the surface of the Al_2_O_3_ NPs. Further, a sturdy absorption band at 1398 cm^−1^ occurs due to the distortion caused by the C–CH_3_, C–C, and C=O bonds. All the samples implicate concerted absorption bands around 3462, 2922, 2850, 2337, 1633, 1385, 1200, and 1118 cm^−1^ as shown in Fig. 2. Broad bands near 3462 and 1633 cm^−1^ are comforted to absorb water molecules' OH-stretching and bending vibrational modes, respectively^27^. Different IR studies of Al_2_O_3_ NPs and spinel metal aluminate nanocomposites observed the water absorption peaks and declared that the high surface area of these materials could result in swift adsorption of water from the atmosphere during pellet squeeze and IR mensuration^27,56^. C–H stretching vibration is represented by small peaks at around 2922 and 2850 cm^−1^^1^. The absorption band at 2337 cm^−1^ is supposedly connected to the entity of CO_2_ on the surface powder, while a small peak at 1385 cm^−1^ can be attributed to the grout nitrogen groups output from the combustion reaction^56^. The peaks in the 1200 cm^−1^ region can be explained as vibrations of the C–C bond, and bands at around 1118 cm^−1^ can be related foremost to oxygen groups with a single C–O bond^28,57^. In the FT-IR spectrum of NiAl_2_O_4_ nanocomposites calcined at 1100 °C, the characteristic bands of NiAl_2_O_4_ appear around 3390, 1629, 725, and 478 cm^−1^, which would affirm the consistence of the NiAl_2_O_4_ aluminate spinel structure in good agreement with the XRD results^29,58^. These bands can be conformed to the uniform stretching, bending, and asymmetric stretching modes of Ni–O, Al–O, and Ni–O–Al bonds at octahedral and tetrahedral sites in M-Al_2_O_4_ spinel^58^. The FT-IR spectrum of CuAl_2_O_4_ nanocomposites calcined at 1100 ℃ confirms the formation of CuAl_2_O_4_ nanocomposites through the strongest four characteristic bands of CuAl_2_O_4_, which are 453, 501, 591, and 640 cm^−1^^24,25,58^. The FT-IR spectrum of ZnAl_2_O_4_ nanocomposites calcined at 1100 °C shows sharp bands between 1634 and 3435 cm^−1^ (C–O and C–C vibration) and 1161 cm^−1^ (NO_3_^−^ vibration), including the three OH vibration bands mentioned above. The bands lying at 1111, 668, and 490 cm^−1^ are comforted by the vibrations of Zn–O, Al–O, and Zn–O–Al bonds in ZnO_4_ tetrahedral and AlO_6_ octahedral groups in the spinel structure^26,52,58^. The spectrum of CoAl_2_O_4_ nanocomposites calcined at 1100 °C is also shown by FT-IR, with sharp bands between 2893 and 3335 cm^−1^ (C–O and C–C vibration) and at 1523 cm^−1^ (NO_3_^−^ vibration). The bands lying at 1113, 670, and 565 cm^−1^ are confined to the vibrations of Co–O, Al–O, and Co–O–Al bonds, which confirmed the formation of CoAl_2_O_4_ nanocomposites.

### Surface morphology and EDX analysis

**Figure 3:**
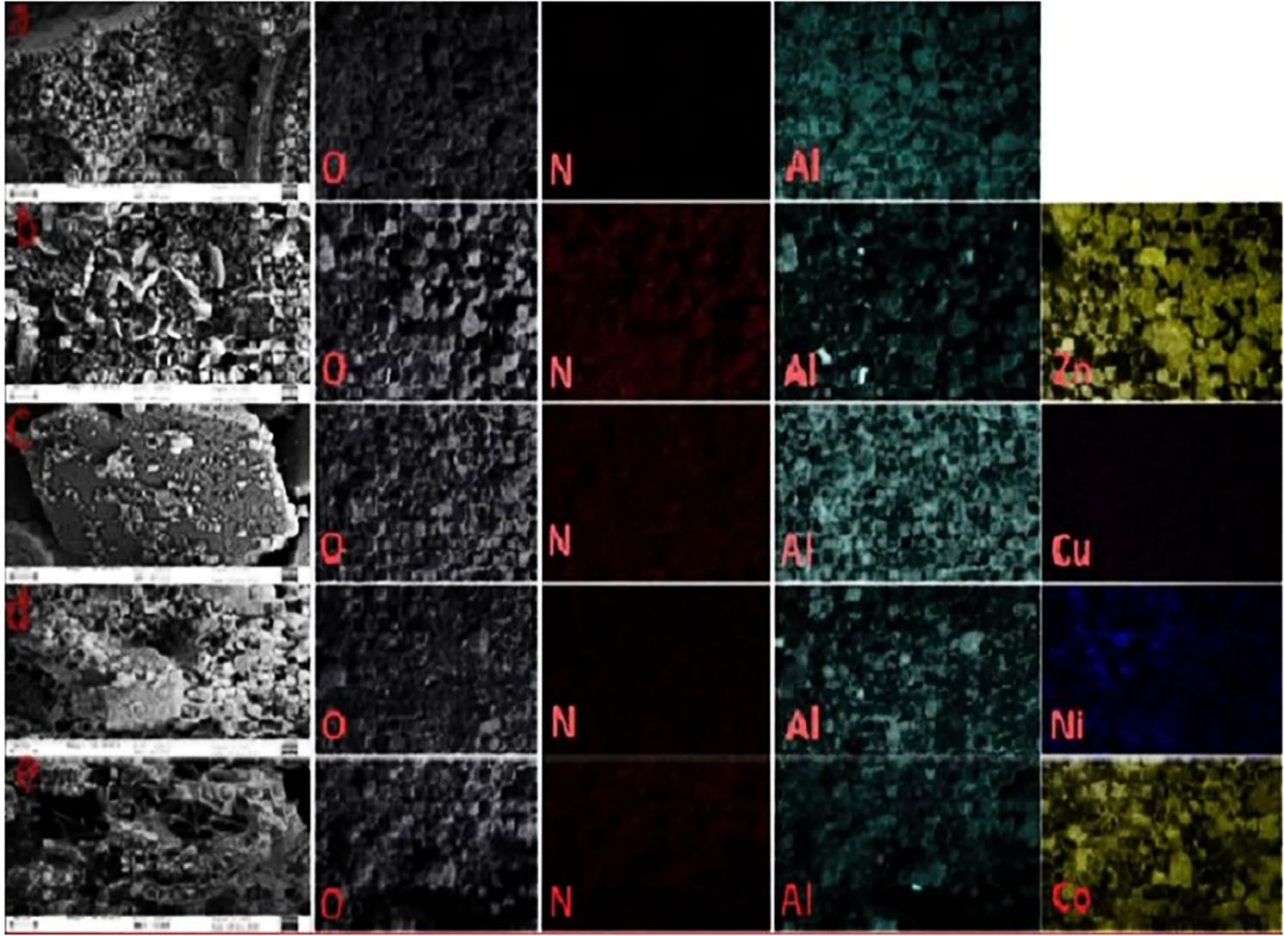
FE-SEM of Al_2_O_3_ NP_s_, ZnAl_2_O_4_, CoAl_2_O_4_, NiAl_2_O_4_, and CuAl_2_O_4_ nanocomposites at 1100 °C.

**Figure 4:**
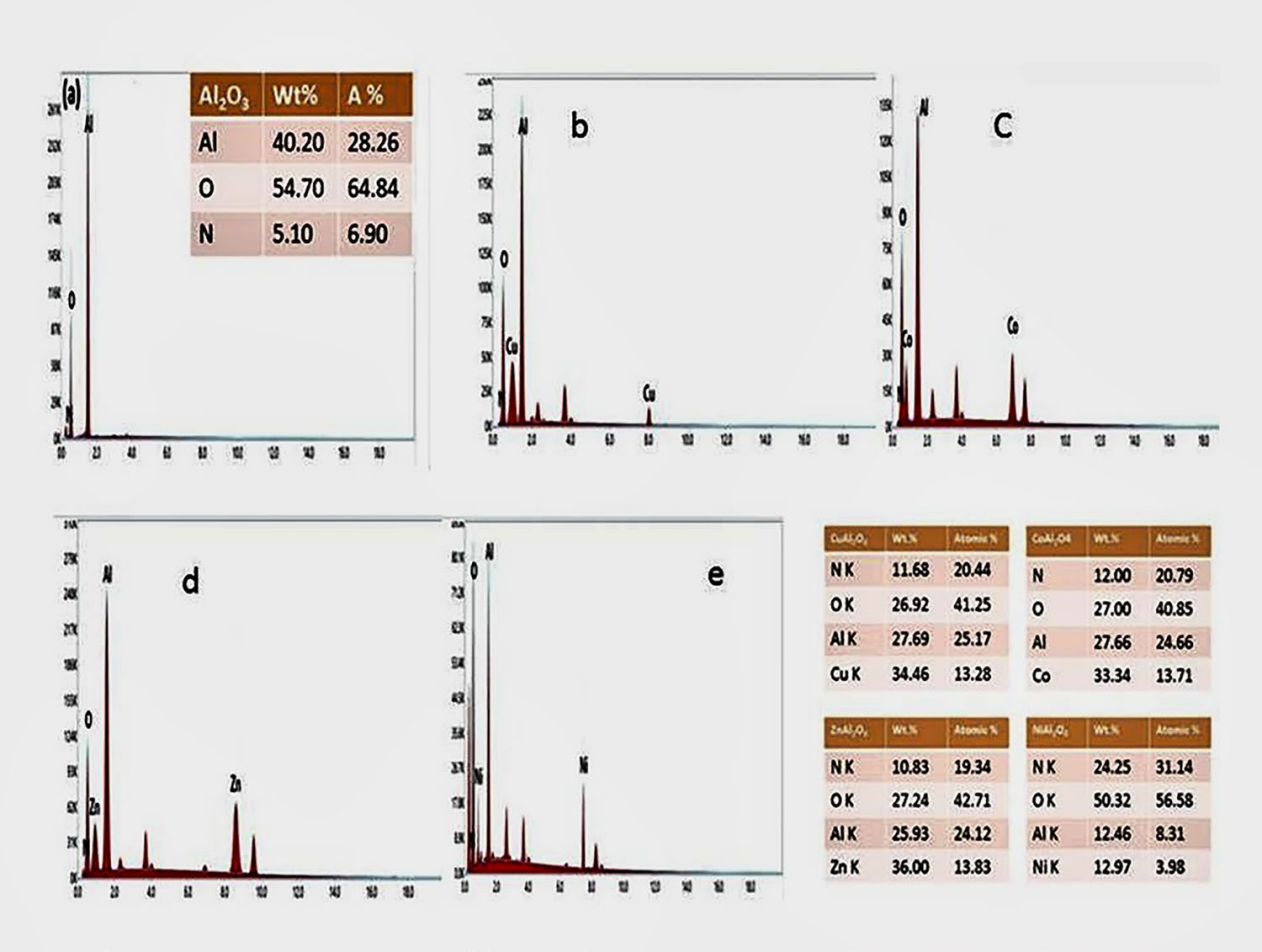
EDX analysis of (**a**)-Al_2_O_3_ NP_s_, (**b**)-CuAl_2_O_4_, (**c**)-CoAl_2_O_4_, (**d**)-ZnAl_2_O_4_ and (**e**)-NiAl_2_O_4_ nanocomposites.

Figure 3 Shows the FE-SEM images and elemental mapping allocation for the Al_2_O_3_ NP_s_, ZnAl_2_O_4_, CuAl_2_O_4_, NiAl_2_O_4_ and CoAl_2_O_4_ nanocomposites calcined at 1100 °C. FE-SEM images of the samples show uniform, spherical, and homogeneous nanoparticles of Al and Zn/Al with mean particle sizes of 29.77 and 44.66 nm, respectively. Figure 3(c) shows the smooth surface and the aggregation of the particles of Cu/Al with a particle size of 33.40 nm. As shown in Fig. 3(d,e), small spherical nanoparticles were neatly packed together with an identical size distribution, and the mean average particle size of NiAl_2_O_4_ and CoAl_2_O_4_ nanocomposites is in the ranges of 18.98–50.07 nm and 40.08–74.53 nm, respectively. In addition, the release of fragile gases such as CO_2_, N_2_, O_2_, and H_2_O during the combustion process should be one of the prime factors that create different pore structures in the ZnAl_2_O_4_, CuAl_2_O_4_, NiAl_2_O_4_, and CoAl_2_O_4_ nanocomposites. This interconnected pore structure is remarkable for catalytic applications. The amount of distribution is dependent on the homogeneity of the size of the particles. The EDX spectrum of Al_2_O_3_ NPs, ZnAl_2_O_4_, CuAl_2_O_4_, NiAl_2_O_4_, and CoAl_2_O_4_ obtained from FE-SEM is shown in Fig. 4(a–e), and the inset table shows the elemental analysis and wt. % of each element. EDX spectra analysis of the atomic% of the elements in ZnAl_2_O_4_, CuAl_2_O_4_, CoAl_2_O_4_, and NiAl_2_O_4_ nanocomposites was found to be 24.12/13.82 = 1.75, 25.17/13.28 = 1.89, 24.66/13.71 = 1.79, 8.31/3.98 = 2.09, respectively, for the best determination with the K-line series. This is in agreement with the results obtained from XRD. Fig. S3 depicts the photography and color of prepared samples as spinel aluminates after 1100 °C annealing.

### Optical properties

**Figure 5:**
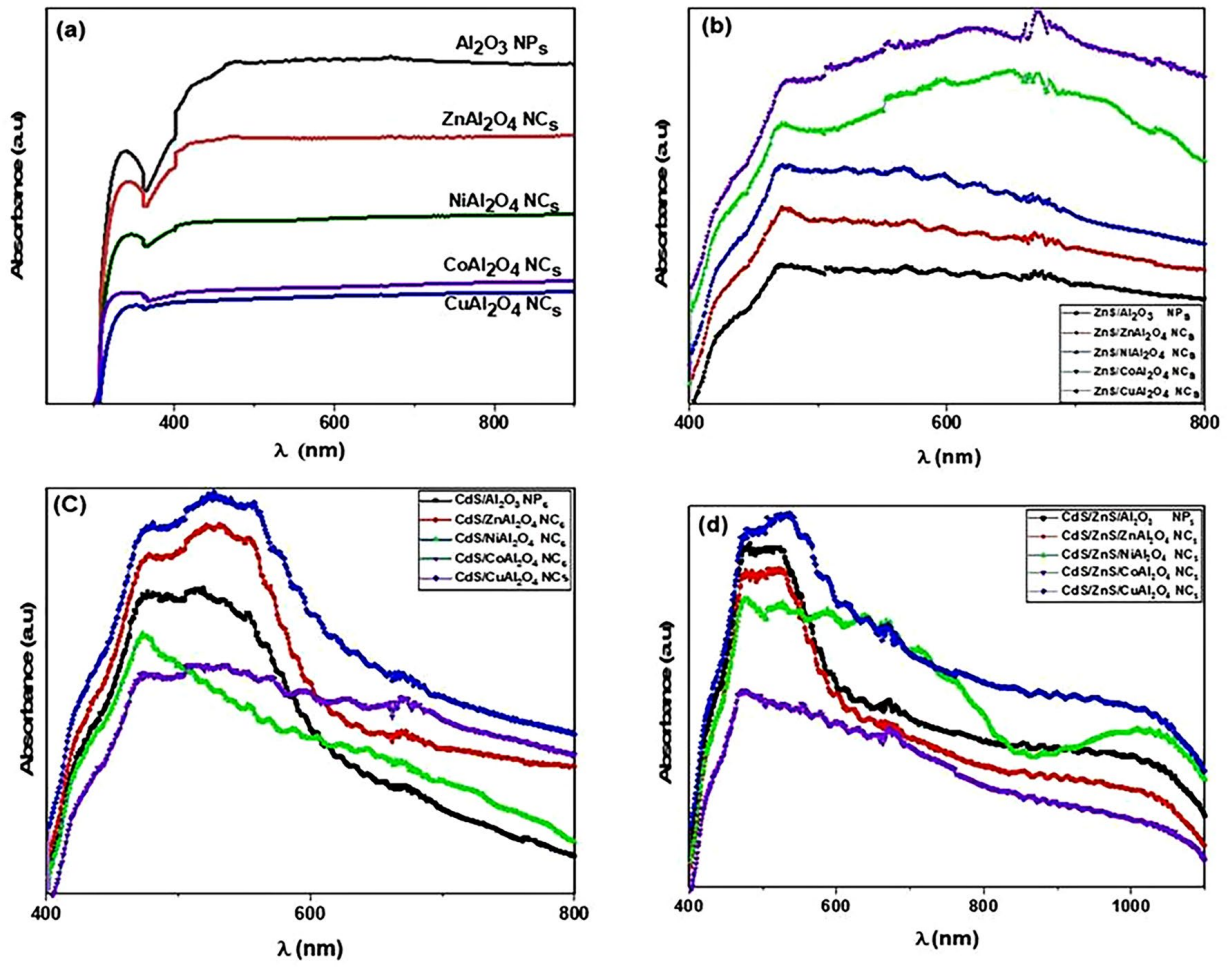
Absorbance spectra of (**a**)-Pure MAl2O4, (**b**)-ZnS/MAl2O4, (**c**)-CdS/MAl2O4 and (**d**)-CdS@ZnS/MAl_2_O_4_.

The absorbance of pure Al_2_O_3_ NPs, ZnAl_2_O_4_, CoAl_2_O_4_, NiAl_2_O_4_, and CuAl_2_O_4_ nanocomposites under UV–Vis irradiation decreases as the wavelength increases. In Fig. 5a, the optical absorbance of the pure Al_2_O_3_ NPs is 1.59% higher than the optical absorbance of ZnAl_2_O_4_, CoAl_2_O_4_, NiAl_2_O_4_ , and CuAl_2_O_4_ nanocomposites, which are (1.4%, 0.7%, 1.06%, and 0.6%), respectively, because the adding of metals like Zn, Co, Ni, and Cu has a high effect on the absorbance and causes a lower shift. Figure 5b shows the absorption spectra of ZnS QDs deposited on these samples to be ZnS/Al_2_O_3_, ZnS/ZnAl_2_O_4_, ZnS/CoAl_2_O_4_, ZnS/NiAl_2_O_4_ and ZnS/CuAl_2_O_4_. The ZnS/CoAl_2_O_4_ sample has a higher absorbance (2.78%) than ZnS/NiAl_2_O_4_, ZnS/CuAl_2_O_4_, ZnS/ZnAl_2_O_4_, and ZnS/Al_2_O_3_, which are (2.65%, 2.52%, 2.39%, and 2.21%) because ZnS QD_s_ are more sensitive to light and rapidly make a shift in absorption edge. Figure 5c shows the absorption spectra of CdS QDs deposited on these samples to be CdS/Al_2_O_3_, CdS/ZnAl_2_O_4_, CdS/CoAl_2_O_4_, CdS/NiAl_2_O_4_, and CdS/CuAl_2_O_4_, where the CdS/CuAl_2_O_4_ sample has a higher absorbance (2.9%) than CdS/Al_2_O_3_, CdS/ZnAl_2_O_4_, CdS/CoAl_2_O_4_, and CdS/NiAl_2_O_4_, which are (2.74%, 2.83%, 2.54%, and 2.63%, respectively). Figure 5d shows the absorption spectra of the hybrid structures of (CdS/ZnS) QDs deposited on these samples to be CdS/ZnS/Al_2_O_3_, CdS/ZnS/ZnAl_2_O_4_, CdS/ZnS/CoAl_2_O_4_, CdS/ZnS/NiAl_2_O_4_, and CdS/ZnS/CuAl_2_O_4_, where the CdS/ZnS/CuAl_2_O_4_ sample has a higher absorbance (2.93%) than CdS/ZnS/Al_2_O_3_, CdS/ZnS/ZnAl_2_O_4_, CdS/ZnS/NiAl_2_O_4_ and CdS/ZnS/CoAl_2_O_4_, which are (2.88%, 2.82%,2.75% and 2.5%, respectively). In addition, comparison between the prepared materials and the same method was done by using the same thickness of these materials.

To know the optical bandgap, we have made an itemized calculation of the band gap using the Tauc formula; (αhν) = A (hυ − Eg) n, where, α is the absorption coefficient, hυ is the photon energy, Eg is the energy gap, and n is the quality of the transitions. The term n is taken as ½ for direct transition and 2 for indirect transition. The optical bandgap energy is investigated by plotting (αhυ) 1/n versus photon energy and drawing the tangent to the curve that intersects with the energy axis at α = 0. The Tauc plot of pure Al_2_O_3_ NPs, ZnAl_2_O_4_, CoAl_2_O_4_, NiAl_2_O_4_, and CuAl_2_O_4_ nanocomposites for direct allowed transition is shown in Fig. S4a. The estimated energy gaps of Al_2_O_3_ NPs, ZnAl_2_O_4_, CoAl_2_O_4_, NiAl_2_O_4_ and CuAl_2_O_4_ nanocomposites are 1.37 eV, 1.5 eV, 1.83 eV, 1.59 eV and 2.07 eV, respectively. The Tauc plots of ZnS/Al_2_O_3_ and ZnS/MAl_2_O_4_ in direct allowed transition are shown in Fig. S4b, and the estimated energy band gaps of ZnS/Al_2_O_3_, ZnS/ZnAl_2_O_4_, ZnS/CoAl_2_O_4_, ZnS/NiAl_2_O_4_, and ZnS/CuAl_2_O_4_ are 1.61 eV, 1.67 eV, and 1.13. The Tauc plots of CdS/Al_2_O_3_ and CdS/MAl_2_O_4_ in direct allowed transition are shown in Fig. S4c, and the estimated energy band gaps of CdS/Al_2_O_3_, CdS/ZnAl_2_O_4_, CdS/CoAl_2_O_4_, CdS/NiAl_2_O_4_, and CdS/CuAl_2_O_4_ are 1.58 eV, 1.38 eV, and 1.33. The Tauc plots of CdS/ZnS/Al_2_O_3_ and CdS/ZnS/MAl_2_O_4_ in direct allowed transition are shown in Fig. S4d, as are the estimated energy band gaps of CdS/ZnS/Al_2_O_3_, CdS/ZnS/ZnAl_2_O_4_, CdS/ZnS/CoAl_2_O_4_, CdS/ZnS/NiAl_2_O_4_, CdS/ZnS/CuAl_2_O_4_ are 1.57 eV, 1.42 eV, 1.64 eV, 1.66 eV, and 1.38 eV, respectively. The absorption coefficient of different materials is calculated by this equation, where (A) is the absorbance of material and (t) is the thickness of it. The thickness of thin films is constant for all materials. The presence of metals like Co, Ni, Zn, and Cu has an effect on the energy gap of materials by decreasing it and contributing to narrowing the band gap of the main semiconductor. All the energy gaps for all materials are listed in the below Table S2.

2 $$ {\upalpha } = \frac{{2.302*{\text{A}}}}{{{\text{t}} }} $$

### Electrochemical impedance spectroscopy

**Figure 6:**
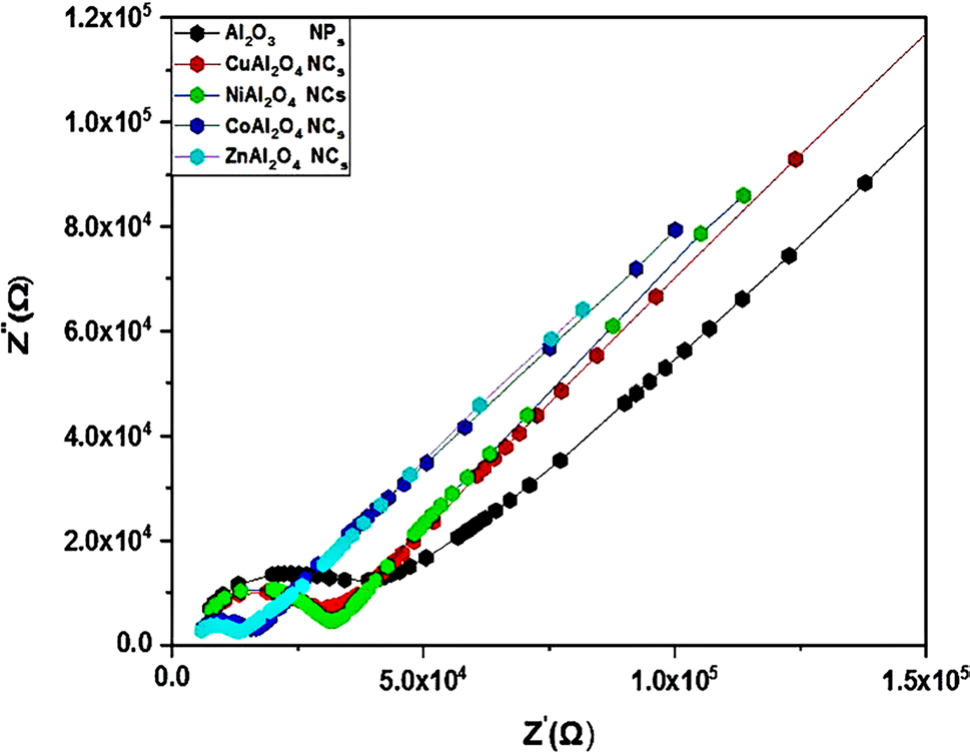
Nyquist plot of EIS spectra of CdS QDs deposited on Al2O3 NPs and MAl_2_O_4_ (M=Cu, Co, Ni, and Zn) nanocomposites as WEs on FTO.

The impedance of Al_2_O_3_ NP_s_, ZnAl_2_O_4_, CoAl_2_O_4_, NiAl_2_O_4_, and CuAl_2_O_4_ nanocomposites with CdS QD_s_ on FTO conductive glass through the dark field was determined by electrochemical impedance spectroscopy (*EIS*) and can be illustrated by the kinetic operation of charge relocated in QDSSCs. Figure 6 shows one semicircle and the second semicircle appears as a straight diffusion line due to the presence of CdS quantum dots in the Nyquist plot of each sample and the same behavior of the samples with CdS@ZnS QD_s_. The data for samples gives the values of charge transport resistance, *Rct for the* first semicircle arc and charge recombination resistance (*Rrec*) for the second semicircle arc at high frequency and low frequency, respectively. The good adhesion of the bond between ZnAl_2_O_4_ nanostructures and the FTO conductive glass substrate pointed to the lower Rct value, which supports more electrons from the external circuit. Charge transfer resistance is of primary significance because it eases the carry of electrons during the catalytic reduction process of an electrolyte. A high electron mobility rate is achieved by a lower value of Rct and causes high electrical outputs, and vice versa. Table 1 shows the relaxation times, diffusion rates, and the Rct value of the ZnAl_2_O_4_ electrode (7.31 Ω.cm^2^) is lower than that of the CuAl_2_O_4_ electrode (7.56 Ω.cm^2^), the NiAl_2_O_4_ electrode (10.94.cm^2^), the Al_2_O_3_ electrode (7.81 Ω.cm^2^), and the CoAl_2_O_4_ electrode (10.5 Ω.cm^2^) with CdS QD_s_^59,60^. Due to the good electrocatalytic attitude of ZnAl_2_O_4,_ it can behave as an efficient WEs catalyst to minimize the oxidized polysulphide electrolyte and high electron mobility rate desired for good photovoltaic performance of QDSSC_s_. Fig. S6 shows the equivalent circuit of QD deposition on nanomaterial preparation.

**Table 1:** Electrochemical Impedance of Al_2_O_3_ NP_s_ and MAl_2_O_4_ with 6 cycles CdS QD_s_ as a photosensitizer.

Sample	R_ct_ Ω.cm^2^*10^4^	R_rec_ Ω.cm^2^*10^5^	C_a_, Farad (F) *10^−10^	Z_f_ (Ω.cm2)	τ_n (ms)_	K_eff_ (ms)^−1^
Al_2_O_3_	7.81	27.25	4.21	2.896	77*10^−3^	12.99
ZnAl_2_O_4_	7.31	11.5	3.66	1.825	11*10^−2^	9.09
NiAl_2_O_4_	10.94	51	5.78	1.424	52*10^−3^	19.23
CuAl_2_O_4_	7.56	22.38	2.38	3.156	87*10^−3^	11.49
CoAl_2_O_4_	10.5	42.44	5.47	2.068	55*10^−3^	18.18

The good adhesion bonding of the nanostructures and the FTO conductive glass substrate enables them to achieve a lower Rct value than NiAl_2_O_4_, which elevates more electrons from the external circuit. The relocating of electrons during the catalytic reduction process of an electrolyte is facilitated by charge transfer resistance. A lower value of *Rct* leads to a high electron mobility rate and causes high electrical outputs, and vice versa. Table 2 shows the relaxation times, diffusion rates, and the Rct value of the NiAl_2_O_4_ electrode (8.11 Ω cm^2^) is lower than that of the CuAl_2_O_4_ electrode (8.31 Ω.cm^2^), the Al_2_O_3_ electrode (44.75 Ω.cm^2^), the ZnAl_2_O_4_ electrode (49.56 Ω.cm^2^), and the CoAl_2_O_4_ electrode (8.63 Ω.cm^2^) with hybrid structures of CdS@ZnS QD_s_^59,60^. Due to the good electrocatalytic attitude of the CuAl_2_O_4,_ it can be behave as an efficient WEs catalyst to minimize the oxidized polysulphide electrolyte and high electron mobility rate desired for good photovoltaic performance of QDSSC_s_. The kinetic operation of charge transport in QDSSCs, electrochemical impedance spectroscopy (EIS), was executed through the exposition of Al_2_O_3_ NPs, ZnAl_2_O_4_, CoAl_2_O_4_, NiAl_2_O_4_, and CuAl_2_O_4_ nanocomposites on FTO conductive glass, where by increasing relaxation time, the diffusion rate is decreased, consequently increasing the power activity of the photoanode in the solar cell, which includes the increasing migration of quantum dots on the semiconductor surface in addition to increasing the motion of free electrons in the cell, as indicated by the obtained data. The equivalent circuit is represented in Fig. S6. From the following equation, the diffusion rate of electrons can be calculated from Eq. (3), the lifetimes (relaxation times) of photogenerated electrons (**τ**_**n**_**)** Eq. (4):

3 $$ {\text{Keff}} = \frac{1}{{{\tau n} }} $$

4 $$ \tau_{n} = 1/2\;\pi f $$

**Table 2:** Electrochemical Impedance of Al_2_O_3_ NP_s_ and MAl_2_O_4_ with 6 cycles CdS/ZnS QD_s_ as a photosensitizer.

Sample	R_ct_ Ω.cm2	R_rec_ Ω.cm2	C_a_, Farad (F)	Z_f_ (Ω.cm2)	τn_(ms)_	K_eff_ (ms)^−1^
Al_2_O_3_	44.75*10^4^	16.69*10^5^	7.18*10^−10^	1.95	81*10^−3^	13.35
ZnAl_2_O_4_	49.56*10^4^	22*10^5^	2.15*10^−9^	1.96	73*10^−3^	13.70
NiAl_2_O_4_	8.11*10^4^	8.25*10^5^	1.22*10^−9^	2.28	96*10^−3^	10.42
CuAl_2_O_4_	8.31*10^4^	10.19*10^5^	1.09*10^−9^	1.87	85*10^−3^	11.76
CoAl_2_O_4_	8.63*10^4^	11.19*10^5^	2.01*10^−9^	1.66	82*10^−3^	12.20

### Photovoltaic performance

Figs. S5a,b show the J-V characteristics of the first systems of QDSSCs composed of Al_2_O_3_ NP_s_, ZnAl_2_O_4_, NiAl_2_O_4_, CoAl_2_O_4_, and CuAl_2_O_4_ nanocomposites as a photoanode with CdS QD_s_ as a photosensitized nanomaterial and P-rGO as a counter electrode, and the second system composed of the same photoanode and counter electrode with hybrid structures of (CdS@ZnS) under one sun illumination (10.4 mW*cm^−2^), respectively. Tables 3 and 4 also summarize the photovoltaic parameters (Jsc, Voc, and η) and cell configuration. The power conversion efficiency of the first system consists of six layers of CdS QD_s_ giving the higher efficiency of ZnAl_2_O_4_ nanocomposites (8.22%) surface based on P-rGO CE compared to the efficiency of Al_2_O_3_ NP_s_, NiAl_2_O_4_, CoAl_2_O_4,_ and CuAl_2_O_4_ (5.77%, 1.84%, 4.15%, and 6.32%), respectively, shown in Table 3, because ZnAl_2_O_4_ has high chemical, mechanical stability, great separation and transfer of light-generated electrons and holes, which corresponds to the current density of 22.86 mA*cm^−2^ and fill factor 0.58. (ZnAl_2_O_4_) nanocomposite with CdS QDs has the highest efficiency. This is due to several reasons:

1. It has the smallest crystallite size as indicated by XRD (Table S1).
2. It has the lowest band-gap energy (Table S2).
3. It has the lowest charge transport resistance (Rct = 7.31*10^4^), charge recombination resistance (Rrce = 11.5*10^5^), diffusion rate (Keff = 9.09), and the highest lifetime of the photogenerated electrons (τ_n_ = 11*10^−2^) as indicated by the electrochemical performance (Table 1).

**Table 3:** Photovoltaic parameter of Al_2_O_3_ NP_s_ and MAl_2_O_4_ with 6 cycles CdS QD_s_ as a photosensitizer with P-rGO as a counter electrode.

Photoanode	Cell configuration	J_sc_ (mA*cm^−2^)	V_oc_ (Volt)	FF	${\varvec{\eta}}$(%)
Al_2_O_3_	FTO/ Al_2_O_3_/CdS/S^−2^*S_n_^−2^/P-rGO/FTO	19.82	0.56	0.55	5.77
ZnAl_2_O_4_	FTO/ZnAl_2_O_4_/CdS/S^−2^*S_n_^−2^/P-rGO/FTO	22.86	0.64	0.58	8.22
NiAl_2_O_4_	FTO/NiAl_2_O_4_/CdS/S^−2^*S_n_^−2^/P-rGO/FTO	12.32	0.35	0.45	1.84
CuAl_2_O_4_	FTO/CuAl_2_O_4_/CdS/S^−2^*S_n_^−2^/P-rGO/FTO	20.72	0.58	0.56	6.32
CoAl_2_O_4_	FTO/CoAl_2_O_4_/CdS/S^−2^*S_n_^−2^/P-rGO/FTO	17.5	0.49	0.5	4.15

**Table 4:** Photovoltaic parameter of Al_2_O_3_ NP_s_ and MAl_2_O_4_ with hybrid structure of (6 cycles CdS/6 cycles of ZnS QD_s_) as a photosensitizer with P-rGO as a counter electrode.

Photoanode	Cell configuration	J_sc_ (mA*cm^−2^)	V_oc_ (Volt)	FF	${\varvec{\eta}}$(%)
Al_2_O_3_	FTO/ Al_2_O_3_/CdS/ZnS/S^−2^*S_n_^−2^/P-rGO/FTO	19.29	0.54	0.56	5.65
ZnAl_2_O_4_	FTO/ZnAl_2_O_4_/CdS/ZnS/S^−2^*S_n_^−2^/P-rGO/FTO	12.5	0.35	0.66	2.79
NiAl_2_O_4_	FTO/NiAl_2_O_4_/CdS/ZnS/S^−2^*S_n_^−2^/P-rGO/FTO	28.22	0.79	0.71	15.14
CuAl_2_O_4_	FTO/CuAl_2_O_4_/CdS/ZnS/S^−2^*S_n_^−2^/P-rGO/FTO	27.43	0.77	0.62	12.55
CoAl_2_O_4_	FTO/CoAl_2_O_4_/CdS/ZnS/S^−2^*S_n_^−2^/P-rGO/FTO	22.68	0.64	0.64	8.86

Zinc oxide has some advantages such that it is a common wide band-gap semiconductor that is now being investigated due to its wide range of applications and adjustable features. The ZnO nanoparticles' wide band-gap semiconductor characteristics are also beneficial in inducing intracellular reactive oxygen species (ROS) production. Conduction electrons (e) and valence holes (h +) in semiconductors have long been employed for photocatalytic oxidation of organic and inorganic contaminants. However, sufficient electrons and holes were routinely generated using UV irradiation and excitation. Even in normal light conditions, great quantities of holes and/or electrons may be accessible in ZnO nanoparticles. P. Sakthivel et al. (2022) show that in the zone wherein light absorption occurs owing to band gap excitation, which reveals that increasing the optical absorption of ZnO by adding defects (such as metal ion doping) to its surface can improve its optical absorption. Aluminum (Al) ions doped on the surface of ZnO nanoparticles boosted the generation of electrons and/or holes in a previous study^49–53^. The second system based on the twelve layers of hybrid structures of CdS@ZnS QD_s_ on the NiAl_2_O_4_ nanocomposite film with P-rGO CE exhibited η of 15.14%. A noticeable improvement in efficiency was observed compared to the cell of Al_2_O_3_ NP_s_, ZnAl_2_O_4_, CoAl_2_O_4_ and CuAl_2_O_4_ (5.65%, 2.79%, 8.86%, and 12.55%), respectively, shown in Table 4. These photovoltaic improvements in the cells are based on the hybrid structures of CdS@ZnS layers on the NiAl_2_O_4_ photoanode with P-rGO CE. The conversion efficiency of the cell *η* can be determined by the equation below:

5 $$ \eta = \frac{Jsc*Voc*FF}{{Pin}} $$

where J_SC_ is the short circuit current density, V_OC_ is the open circuit voltage, FF is the fill factor, and Pin is the power intensity of the incident light. From the J-V curves, FF values can be determined.

## Conclusion

Aluminum Oxide nanoparticles and spinel metal aluminate nanocomposites have been successfully prepared by the auto-combustion sol–gel method. The electrochemical performance of metal aluminates Al_2_O_3_ NP_s_ and MAl_2_O_4_ (M=Zn, Co, Ni, and Cu) is greatly affected by the loading of different quantum dots (CdS) and hybrid structures (CdS/ZnS). The hybrid structures of (CdS/ZnS) on metal aluminates showed a high rendering as photoanodes in QDSSC_s_, whereby by increasing the life time of quantum dots, the diffusion rate is decreased and the (PCE) increased. The highest activity was 15.14% in the cell containing NiAl_2_O_4_ spinel with (CdS/ZnS) QD_s_. The EIS analysis showed that NiAl_2_O_4_ has the lowest diffusion rate, Keff, and the highest electron life time, *τ*_*n*_*.*

## Supplementary Information

Supplementary Information 1.

Supplementary Information 2.

Supplementary Information 3.

Supplementary Information 4.

Supplementary Information 5.

Supplementary Information 6.

Supplementary Information 7.

Supplementary Information 8.

## Data Availability

All data generated or analysed during this study are included in this published article (and its Supplementary Information files).
