# Supplementary material for: Synthesis and electrochemical performance of α-Al2O3 and M-Al2O4 spinel nanocomposites in hybrid quantum dot-sensitized solar cells
Source: Sci Rep. 2022 Oct 11;12:17009. doi: 10.1038/s41598-022-21186-4 (PMC9554019; doi:10.1038/s41598-022-21186-4)
Supplement: Supplementary file 7 — Supplementary Information 7. [file 41598_2022_21186_MOESM7_ESM.docx]

**TableS 1.** Average crystallite size (D), interplanar distance (d), thickness (t), and band gap value (Eg) of single-phase Al_2_O_3_ NP_s_, ZnAl_2_O_4_, CuAl_2_O_4_, NiAl_2_O_4_ and CoAl_2_O_4_ nanocomposites.

| *E_g_ (e.V)* | *Thickness (mm)* | *Crystallite size; D (nm)* | *d_(311)_ A^◦^* | *Sample* |
| --- | --- | --- | --- | --- |
| 1.37 | **0.053** | **28.9** | **2.085** | **Al_2_O_3_ NP_s_** |
| 1.5 | **0.053** | **17.49** | **2.477** | **ZnAl_2_O_4_ NC_s_** |
| 2.07 | **0.053** | **24.64** | **2.087** | **CuAl_2_O_4_ NC_s_** |
| 1.59 | **0.053** | **20.7** | **2.415** | **NiAl_2_O_4_ NC_s_** |
| 1.83 | **0.053** | **12.37** | **2.438** | **CoAl_2_O_4_ NC_s_** |

| Sample | E_g_ of Pure materials (eV) | E_g_ of materials with ZnS QD_s_ (eV) | E_g_ of materials with CdS QD_s_ (eV) | E_g_ of materials with CdS@ZnS QD_s_ (eV) |
| --- | --- | --- | --- | --- |
| Al_2_O_3_ | **1.37** | **1.61** | **1.58** | **1.57** |
| ZnAl_2_O_4_ | **1.5** | **1.67** | **1.38** | **1.42** |
| CoAl_2_O_4_ | **1.83** | **1.13** | **1.33** | **1.64** |
| NiAl_2_O_4_ | **1.59** | **1.43** | **1.69** | **1.66** |
| CuAl_2_O_4_ | **2.07** | **1.58** | **1.44** | **1.38** |

**Table S2.** The bandgap energy of Al_2_O_3_ NPs and spinel nanocomposites with ZnS QD_s_, CdS QD_s_, and CdS@ZnS QD_s_.
