# Supplementary material for: Synthesis and electrochemical performance of α-Al2O3 and M-Al2O4 spinel nanocomposites in hybrid quantum dot-sensitized solar cells
Source: Sci Rep. 2022 Oct 11;12:17009. doi: 10.1038/s41598-022-21186-4 (PMC9554019; doi:10.1038/s41598-022-21186-4)
Supplement: Supplementary file 8 — Supplementary Information 8. [file 41598_2022_21186_MOESM8_ESM.docx]

**Figure Captions supplementary:**

**Figure S1.** A schematic diagram depicts the synthesis of P-rGO using a modified Hummers method.

**Figure S2.** A schematic diagram depicts the structure of QDSSC_s_.

**Figure S3.** Photographs of Al_2_O_3_ NP_s_, NiAl_2_O_4_, ZnAl_2_O_4_, CoAl_2_O_4_ and CuAl_2_O_4_ NC_s_ calcined at 1100^o^C and P-rGO.

**Figure S4.** Optical band gap of (**a**) - pure MAl_2_O_4_, (**b**)-ZnS/MAl_2_O_4_, (**c**)-CdS/MAl_2_O_4_ and (**d**) - CdS@ZnS/MAl_2_O_4_.

**Figure S5 a.** J-V curves of Al_2_O_3_ NP_s_, ZnAl_2_O_4_, CoAl_2_O_4_, NiAl_2_O_4_ and CuAl_2_O_4_ NC_s_ electrodes based on CdS QD_s_ and P-rGO CE under one sun illumination

**Figure S5 b.** J-V curves of Al_2_O_3_ NP_s_, ZnAl_2_O_4_, CoAl_2_O_4_, NiAl_2_O_4_ and CuAl_2_O_4_ NC_s_ electrodes based on hybrid structure of CdS/ZnS QD_s_ and P-rGO CE under one sun illumination.

**Figure S6.** Equivalent circuit proposed to fit the EIS data for CdS QDs and CdS@ZnS QDs
